# Recurrent Chromosome 16p13.1 Duplications Are a Risk Factor for Aortic Dissections

**DOI:** 10.1371/journal.pgen.1002118

**Published:** 2011-06-16

**Authors:** Shao-Qing Kuang, Dong-Chuan Guo, Siddharth K. Prakash, Merry-Lynn N. McDonald, Ralph J. Johnson, Min Wang, Ellen S. Regalado, Ludivine Russell, Jiu-Mei Cao, Callie Kwartler, Kurt Fraivillig, Joseph S. Coselli, Hazim J. Safi, Anthony L. Estrera, Suzanne M. Leal, Scott A. LeMaire, John W. Belmont, Dianna M. Milewicz, GenTAC Investigators

**Affiliations:** 1 Department of Internal Medicine, University of Texas Health Science Center at Houston, Houston, Texas, United States of America; 2 Department of Molecular and Human Genetics, Baylor College of Medicine, Houston, Texas, United States of America; 3 Division of Cardiothoracic Surgery, Michael E. DeBakey Department of Surgery, Baylor College of Medicine, Houston, Texas, United States of America; 4 Cardiovascular Surgery, Texas Heart Institute at St. Luke's Episcopal Hospital, Houston, Texas, United States of America; 5 Department of Cardiothoracic and Vascular Surgery, University of Texas Health Science Center at Houston, Houston, Texas, United States of America; 6 Internal Medicine Service, Texas Heart Institute at St. Luke's Episcopal Hospital, Houston, Texas, United States of America; The Children's Hospital of Philadelphia, United States of America

## Abstract

Chromosomal deletions or reciprocal duplications of the 16p13.1 region have been implicated in a variety of neuropsychiatric disorders such as autism, schizophrenia, epilepsies, and attention-deficit hyperactivity disorder (ADHD). In this study, we investigated the association of recurrent genomic copy number variants (CNVs) with thoracic aortic aneurysms and dissections (TAAD). By using SNP arrays to screen and comparative genomic hybridization microarrays to validate, we identified 16p13.1 duplications in 8 out of 765 patients of European descent with adult-onset TAAD compared with 4 of 4,569 controls matched for ethnicity (*P* = 5.0×10^−5^, OR = 12.2). The findings were replicated in an independent cohort of 467 patients of European descent with TAAD (*P* = 0.005, OR = 14.7). Patients with 16p13.1 duplications were more likely to harbor a second rare CNV (*P* = 0.012) and to present with aortic dissections (*P* = 0.010) than patients without duplications. Duplications of 16p13.1 were identified in 2 of 130 patients with familial TAAD, but the duplications did not segregate with TAAD in the families. *MYH11*, a gene known to predispose to TAAD, lies in the duplicated region of 16p13.1, and increased *MYH11* expression was found in aortic tissues from TAAD patients with 16p13.1 duplications compared with control aortas. These data suggest chromosome 16p13.1 duplications confer a risk for TAAD in addition to the established risk for neuropsychiatric disorders. It also indicates that recurrent CNVs may predispose to disorders involving more than one organ system, an observation critical to the understanding of the role of recurrent CNVs in human disease and a finding that may be common to other recurrent CNVs involving multiple genes.

## Author Summary

Thoracic aortic aneurysms and acute aortic dissections (TAAD) have ranked as high as the fifteenth leading cause of death in the United States. TAAD can be inherited in families in an autosomal dominant manner, and mutations in *ACTA2* and *MYH11*, genes encoding two major components of the smooth muscle contractile unit, are responsible for approximately 15% of familial TAAD. However, the majority of patients with TAAD do not have an identified syndrome or family history of aortic disease, and genetic factors predisposing to these sporadic cases have not been identified. To determine whether recurrent genomic copy number variants (CNVs) contribute to TAAD pathogenesis, we screened 765 patients with adult-onset TAAD for CNVs and identified recurrent 16p13.1 duplications in 1% of TAAD cases compared with 0.09% of controls. The 16p13.1 duplication involves 9 genes, including *MYH11*. This recurrent duplication of 16p13.1 has also been determined to be associated with neuropsychiatric conditions, specifically schizophrenia and attention-deficit hyperactivity disorder. Our study suggests that recurrent duplications of 16p13.1 confer a risk for both neuropsychiatric diseases and TAAD, a finding that may be common to other recurrent CNVs involving multiple genes.

## Introduction

Recurrent copy number variants (CNVs) in the human genome occur in areas of the genome prone to non-allelic homologous recombination (NAHR) due to unequal crossover between large regions of highly identical segmental duplications (>10 Kb in length with >90% sequence identity) [1]–[3]. The short arm of chromosome 16 contains an unusually high number of interspersed segmental repeats, which lead to recurrent deletions and duplications of discrete regions such as 16p13.1. Deletions of 16p13.1 vary in size but typically involve 14.7 Mb to 16.3 Mb and have been described in a variety of complex mental disorders such as autism, mental retardation, schizophrenia, attention-deficit hyperactivity disorder (ADHD), and epilepsy [1], [4]–[8]. The prevalence of reciprocal duplications of 16p13.1 is significantly increased in patients with schizophrenia and ADHD [9], [10]. Duplications or deletions of 16p13.1 can be inherited in families or occur *de novo*. CNVs involving 16p13.1 are also found in normal controls, which raise the question as to what determines the pathogenicity of these CNVs.

Aortic aneurysms involving the ascending thoracic aorta predispose to acute aortic dissection, and deaths due to aortic dissections have ranked as high as the 15^th^ leading cause of death by the Center of Disease Control [11], [12]. Although hypertension and bicuspid aortic valve (BAV) are both risk factors for thoracic aortic aneurysms and dissections (TAAD), one in five patients has one or more affected relatives [13], [14]. Thoracic aortic disease can be a complication of genetic syndromes resulting from a single gene mutation, such as Marfan syndrome (MFS) [15], but more commonly a predisposition for aortic disease is inherited in families as an autosomal dominant condition without syndromic features, termed familial thoracic aortic aneurysms and dissections (FTAAD) [16]. A number of mutant genes have been identified that predispose to FTAAD, including *MYH11* (MIM 160745), *ACTA2* (MIM 100678), *TGFBR2* (MIM 190182), *TGFBR1* (MIM 190181) and *MYLK* (MIM 600922) [17]–[21]. *MYH11* mutations, which include missense mutations and in-frame splicing errors and deletions, are responsible for 1% of FTAAD and are found in families presenting with aortic disease and patent ductus arteriosus. The majority of patients who have thoracic aortic aneurysms and dissections do not have an identified syndrome or family history of aortic disease.

Genetic factors predisposing to these sporadic thoracic aortic aneurysms and dissections (STAAD) have not been identified. We previously reported the first genome-wide copy number analysis of STAAD patients using single nucleotide polymorphism arrays [22]. Gene ontology and network analysis demonstrated that rare CNVs in STAAD patients were enriched for genes that regulate vascular SMC adhesion and contractility. One of the most common recurrent STAAD-associated CNVs involved large duplications of chromosome 16p13.1, and we sought to validate and further characterize this duplication in STAAD patients in this study.

## Results

### Duplications of 16p13.1 in STAAD patients

**Figure 1:**
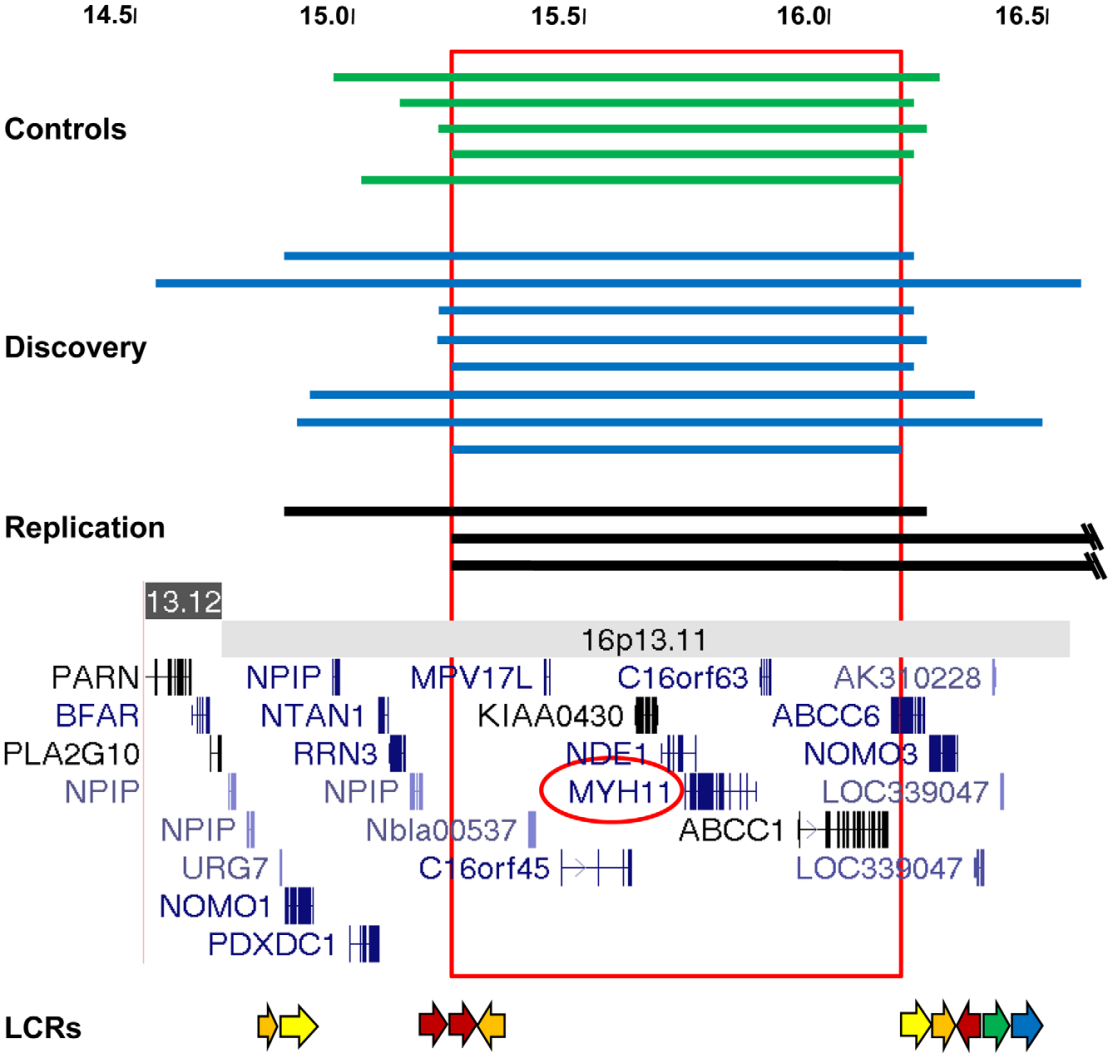
Characterization of 16p13.1 duplications in individuals with thoracic aortic aneurysms and dissections. (A) The extent of 16p13.1 duplications in controls (green), the discovery cohort (blue) and the replication cohort (black) is shown. The scale is in megabases. The common duplicated region that is spanned by all CNVs is boxed. Below is a schematic of the 16p13.1-p12.3 region, which includes the location of genes and low-copy repeats (LCR, arrows).

Single nucleotide polymorphism (SNP) array data (Illumina Human CNV370-Quad BeadChip) obtained from 765 unrelated STAAD patients of European descent over the age of 30 years (STAAD-1 cohort) were analyzed for CNVs and compared with CNVs identified from SNP array data from 4569 ethnically matched controls (Table S1) as previously reported [22]. Comparing the STAAD-1 cases and 4569 controls from 4 dbGAP control datasets revealed only weak evidence of population substructure (λ = 1.05). We identified large heterozygous 16p13.1 duplications of variable size in 8 of 765 (1.0*%*) STAAD patients compared with 4 duplications of 16p13.1 involving the same region in 4569 (0.09%) ethnically matched controls (Table 1, Table 2, and Table S2; Figure 1, Figures S1 and S2). Thus, 16p13.1 duplications were significantly enriched in patients with STAAD (Fisher's exact *P* = 5.0×10^−5^, odds ratio (OR) = 12.2, 95% confidence interval (CI) = 3.2–54.8). We validated the 16p13.1 duplications with a second independent assay, a customized oligonucleotide array that targeted the common duplicated interval between 14.0 and 17.7 Mb of 16p13.1 (Figure S3). The 16p13.1 duplications ranged in size from 0.84 to 2.1 Mb and encompassed a common 0.84 Mb genomic interval from 14.6 to 16.7 Mb on chromosome 16p13.1. One patient with segmental uniparental isodisomy of 16p (start 0, end 30888403) was not included in our analysis. The duplicated region contained between 9 and 18 genes, and 9 genes were duplicated in all patients: *MPV17L*, *C16orf45*, *KIAA0430*, *NDE1*, *MYH11*, *C16orf63*, *ABCC1*, *ABCC6*, and *NOMO3*. The variable size of the duplications and haplotype analysis of flanking SNPs indicate that the duplications in these patients are unique and independent events. No deletions of 16p13.1 were identified in STAAD-1 patients.

**Table 1:** Frequency of 16p13 duplications in cases and controls.

	Cases		Controls		Significance	
	16p13 dup	Total	16p13 dup	Total	OR (95% CI)	*P* value
**Discovery**	8	765	4	4569	12.2 (3.2–54.8)	5.0×10^−5^
**Replication**	5	467	1	1361	14.7 (1.6–694)	0.005
**Combined**	13	1232	5	5930	12.6 (4.2–45.3)	3.97×10^−7^
**Published Controls** a			32	34421	11.3 (5.4–22.3)	2.77×10^−9^
**Total**	13	1232	37	40351	10.7 (5.1–21.1)	1.4×10^−8^

**^a^**Data on published controls is cited from [8].

**Table 2:** Clinical characteristics of patients with 16p13.1 duplications.

ID	Cohort	Presentation	Age atOnset(Years)	Gender	Method	CNV Start (kb)	CNVEnd(kb)	Length(kb)	BAV	HTN (no. of meds)	Second CNV	Other conditions	Neuropsych
521044	STAAD	Type A	56	F	A, C	15,370	16,216	846	+	+ (0)	+		-
4808142	STAAD	Type A	42	M	A, C	14,977	16,434	147	−	−			Anxiety disorder (med)
MG4956	STAAD	Type A	67	M	A, C	14,897	16,464	157	−	+ (1)		CAD, AODM	-
MG5041	STAAD	Type A	60	M	A, B, C	14,617	16,740	213	−	+ (1)	+	Colon cancer	-
MG5609	STAAD	Type A	50	M	A, B , C	15,272	16,371	109	+	+ (1)		Stroke	-
MG3392	STAAD	Type B	53	M	A, B, C	15,323	16,371	104	−	+ (3)		Dilated asc aorta	-
MG8233	STAAD	Type A	61	M	A, C	15,370	16,216	846	−	+ (2)	+	CAD, CHF, stroke, testicular cancer	-
MG7647	STAAD	Type B	59	M	A, C	14,809	16,570	1,761	−	+ (3)		COPD, AODM	-
MG6343	STAAD	Type B	41	M	A, B, C	14,897	16,528	1,631	−	+ (0)		Pacemaker	-
MG4890	STAAD	Type B	40	M	B, C	NA	NA	NA	−	+ (0)			Alcoholic
MG9973	BAV/TAAD	Asc aneurysm	64	F	A, C	14,950	16,365	1,415	+	−		UNK	UNK
MG6982	FTAAD-TAA337	Type A	51	M	A, C	15,387	18,579	3,192	−	−		Rectal cancer(41 yrs)	-
MG9076	FTAAD-TAA499	Asc aneurysm	45	M	A, C	15,387	18,174	2.787	−	−			-

STAAD: sporadic thoracic aortic aneurysm and dissection (TAAD); FTAAD; familial TAAD; Asc: ascending; Methods of detection of the 16p13.1 duplication include the following: (A) Illumina SNP array; (B) Agilent oligonucleotide comparative genome hybridization (CGH) array; (C) Quantitative real-time PCR of probes located throughout the duplicated region; CNV start and end, beginning and end of CNV based on NCBI Build 36.1 of human reference sequence; Length: size of CNV; BAV; bicuspid aortic valve; CAD, coronary artery disease; AODM, adult onset diabetes mellitus; CHF, congestive heart failure; UNK, unknown; Neuropsych; presence of neuropsychiatric conditions; med, on medication.

Interestingly, patients with 16p13.1 duplications were significantly more likely than controls to harbor a second rare CNV. Three patients with 16p13.1 duplications harbored additional CNVs that were unique to STAAD patients and not found in 4569 unaffected controls. In comparison, only 7 of 134 STAAD patients without 16p13.1 duplications had two or more rare CNVs not found in controls (Fisher's exact *P* = 0.012) (Table S3).

**Figure 2:**
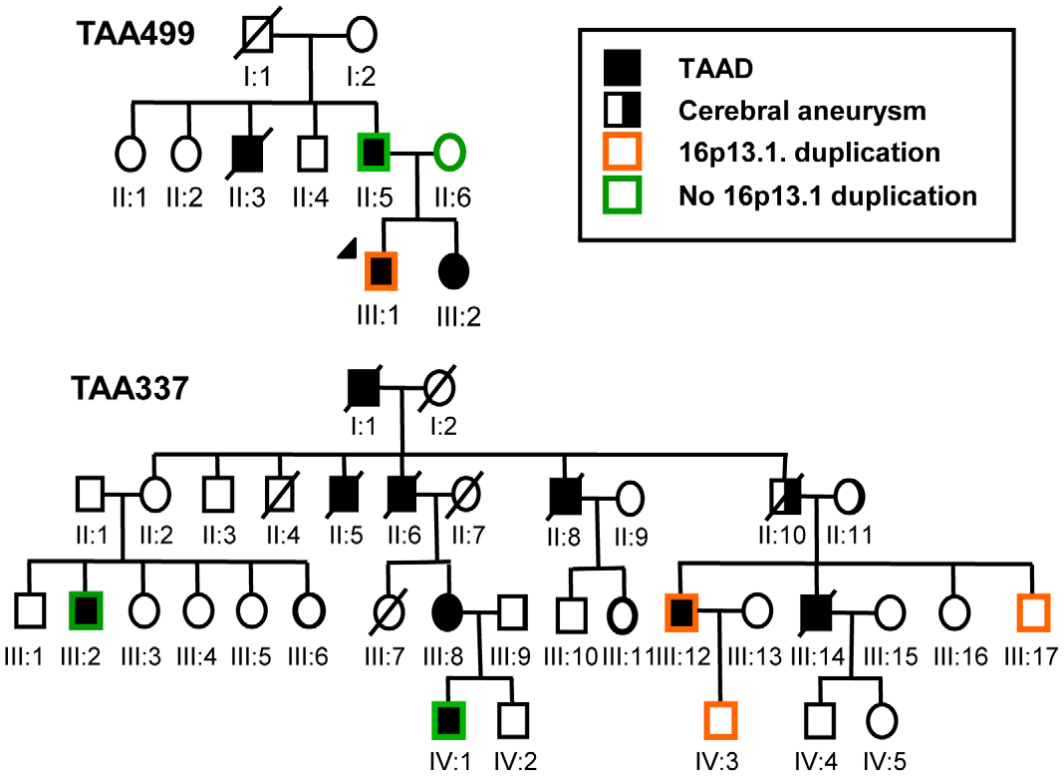
Segregation of the 16p13.1 chromosomal duplication in patients with familial inheritance of thoracic aortic aneurysms and dissections. Circles indicate females; squares indicate males. Pedigrees of families TAA337 and TAA499 are shown, and the legend indicates the diseases and the presence or absence of the 16p13.1 duplication in the family members.

To replicate the association of 16p13.1 duplications with STAAD, quantitative PCR (Q-PCR) assays were designed to assess the number of alleles at the 16p13.1 locus in genomic DNA. Since *MYH11* was common to all the identified duplications and the best candidate for the dosage-sensitive gene conferring increased aortic disease risk, probes were designed for exons 2, 19 and 27 of *MYH11* for this assay (Figure S4), along with probes for other genes, specifically *PDXDC1*, *C16orf45* and *ABCC1* (Figure S1, Table S4). Quantification of copy numbers for these genes using Q-PCR coincided with the copy number derived from the Illumina SNP array analysis of the 8 original samples, as well as 15 samples without 16p13.1 duplications (Figure 1; Figures S1, S2, S3). Using the Q-PCR assay, we identified two 16p13.1 duplications in 242 STAAD patients of European descent who were recruited using the same clinical criteria as the STAAD-1 cohort (STAAD-2 cohort; Table S1). Additionally, a cohort of 95 patients of European descent with bicuspid aortic valves and ascending aortic aneurysms or aortic dissections (BAV/TAAD cohort) was obtained from the GenTAC registry and screened for 16p13.1 duplications (Figure 1 and Table S5). One BAV/TAAD patient was identified with a 16p13.1 duplication. To determine if 16p13.1 duplications caused an inherited predisposition to TAAD, we screened 130 unrelated affected probands with familial TAAD and without identified causative mutations (FTAAD cohort) [17]. In family TAA499, the 16p13.1 a *de novo* duplication in the affected proband and did not segregate with aortic disease (Figure 2). In family TAA337, the duplication 16p13.1 was inherited but failed to segregate with thoracic aortic disease. In total, the replication cohort consisted of 466 patients. We identified 5 patients (1.1%) with 16p13.1 duplications in comparison with 1 of 1361 controls (Table 1, *P* = 0.005, OR = 14.7, 95% CI = 1.6–694), including no duplications identified in 521 local controls of European descent and one of 840 SNP genotypes in a dbGAP dataset derived from SNP array data on controls of European descent without known vascular disease. A SNP array platform (Illumina Human 660W-Quad Beadchip) was used to confirm the duplications identified by Q-PCR in the FTAAD and BAV/TAAD cohorts. Combining the data from all cohorts (1232 patients and 5930 controls) resulted in a highly significant association between TAAD and duplications of 16p13.1 (*P* = 3.97×10^−7^, OR = 12.6, 95% CI = 4.2–45.3).

### Phenotype, pathology, and MYH11 expression levels in patients with 16p13.1 duplications

Analysis of clinical data from the 16p13.1 duplication STAAD patients revealed that all 11 patients from the STAAD cohorts with 16p13.1 duplications presented with aortic disease that had progressed to aortic dissection involving either the ascending (type A) or descending (type B) aorta (Table 2). The sizes of the ascending aorta at the time of type A dissections were noted to be between 4.5–5.0 cm, which is smaller than aortic diameter triggering referral for surgical repair (5.0–5.5 cm). Therefore, unlike TAAD patients without duplications who more often harbor clinically stable aortic aneurysms, patients with duplications were more likely to dissect (*P* = 0.010). Importantly, review of the medical records of the 11 STAAD and 2 FTAAD patients with 16p13.1 duplications found no evidence of autism, developmental delay, ADHD, schizophrenia, or congenital anomalies including patent ductus arteriosus, although one patient was on medication for an anxiety disorder, while another patient abused alcohol (information not available from GenTAC patients, Table 2).

**Figure 3:**
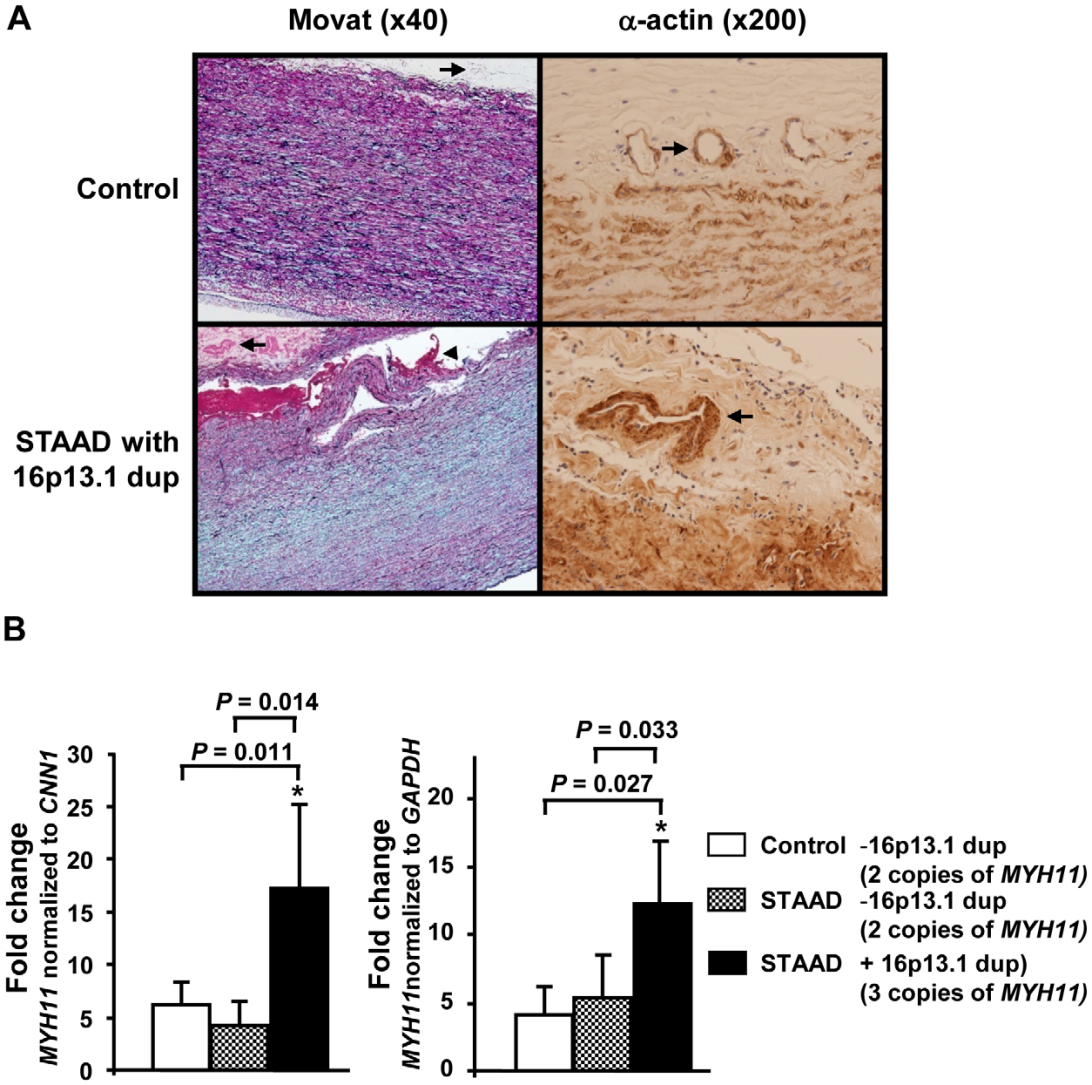
Pathological abnormalities and *MYH11* expression levels in aortic tissue associated with 16p13.1 duplication in patients with staad. (A) Movat staining of aortic media from a 16p13.1 duplication patient shows medial degeneration characterized by proteoglycan accumulation (stained blue), loss and fragmentation of elastic fibers (stained black) and an acute aortic dissection (arrowhead) when compared with a control aorta. Some of the arteries in the vasa vasorum of the patients with 16p13.1 duplication showed increased size and thickness (arrows). Smooth muscle cell (SMC) alpha-actin staining of the vasa vasorum indicated that increased thickness of vasa vasorum was due to increased SMCs in the medial layer. (B) Quantitative real-time PCR assays (Q-PCR) of *MYH11* expression levels in ascending aortic tissues from STAAD patients with and without 16p13.1 duplications and control aortic tissues indicated that *MYH11* message levels were significantly increased in patients with 16p13.1 duplication (n = 4), compared to those of patients without 16p13.1 duplication (n = 6) or to controls (n = 5). The relative *MYH11* mRNA expression was determined by Q-PCR and normalized to either calponin-1 (*CNN1*) or *GAPDH*.

Assessment of the ascending aortic pathology of patients with 16p13.1 duplications identified medial degeneration of the aorta characterized by loss of elastic fibers and proteoglycan accumulation (Figure 3A). Fibromuscular dysplasia was evident in arteries of the *vasa vasorum*, a finding previously observed in aortas from patients with *MYH11* mutations [23]. Since *MYH11* lies within the duplicated 16p13.1 region, *MYH11* expression was assessed using ascending aortic tissue from STAAD patients with the 16p13.1 duplications. *MYH11* expression levels were increased in the RNA from 16p13.1 duplication aortas compared with aortas from patients without the duplication and age-matched controls, using either smooth muscle (SM) calponin 1 (*CNN1*, a SM-specific gene; *P* = 0.011 and 0.014, respectively) or glyceraldehyde-3-phosphate dehydrogenase (*GAPDH*,) expression as an internal control (*P* = 0.033 and 0.027, respectively; Figure 3B). *MYH11* expression levels in ascending aortic tissue were not significantly different between STAAD patients' aortas without the 16p13.1 duplication and control aortas.

## Discussion

Here we report a twelve-fold overrepresentation of chromosome 16p13.1 duplications in patients with thoracic aortic disease (1.06% versus 0.09% in controls), indicating greater enrichment of this duplication in thoracic aortic disease than the three-fold overrepresentation previously identified in schizophrenic patients and the five-fold increase in attention-deficit hyperactivity disorder [10]. Note that the frequency of the 16p13.1 duplication in European controls or controls of European descent of 0.09% was identical between our study and both the schizophrenia and attention-deficit hyperactivity disorder studies [4]. Although the 16p13.1 duplications vary in size, the duplicated regions associated with these three disorders overlap. Therefore our data support a stronger predisposition for thoracic aortic disease than for neuropsychiatric disease with 16p13.1 duplications. In contrast with other contiguous gene defects, such as the 1q21.1 deletion syndrome, we found that 16p13.1 duplications are associated with an adult-onset cardiovascular disorder in the absence of significant neuropsychiatric abnormalities [24], [25]. These data indicate that CNVs may be associated with non-overlapping phenotypes that affect more than one organ system, an observation critical to our understanding of the role of recurrent CNVs in human disease and a finding that may be common to other recurrent CNVs involving multiple genes.

Significant associations were recently reported between neuropsychiatric disorders and rare but recurrent deletions and duplications involving the short arm of chromosome 16. These studies used cohorts of patients with and without a family history of the disease, similar to the study reported here [9], [26]. Although our data on segregation of the 16p13.1 duplications were limited in this study, both inherited and *de novo* duplications were identified in FTAAD patients. As in prior studies of duplications or deletions involving this region, we found that the association between 16p13.1 duplications and STAAD is moderated by decreased penetrance, as illustrated by the identification of these CNVs in controls and unaffected family members. Among 16p13.1 duplication carriers, the risk for thoracic aortic disease is relatively higher than the risk for schizophrenia, but less than the risk associated with single gene mutations that cause familial TAAD. We hypothesize that the duplications may act to modify the age of onset and dissection risk in families with single gene mutations. At the same time, the effect of 16p13.1 CNVs is substantially greater than the typical effect size of common variants identified in genome-wide association studies. Although we cannot exclude the possibility that the 16p13.1 leads to another, unidentified trait that increases the risk for TAAD, our findings suggest that rare CNVs with moderate effects are an important part of the allelic spectrum that contributes to the risk for TAAD.

This is the first report of an association between a recurrent CNV and an adult-onset vascular disease, although the assessment of CNVs in vascular diseases has been limited [22], [27], [28]. In addition to the increased frequency of 16p13.1 duplications in TAAD in this study, we also identified increased numbers of rare CNVs in TAAD patients compared to controls, as well as a greater prevalence of additional rare CNVs in TAAD patients with 16p13.1 duplications [22]. In addition, ontology analysis of the rare CNVs identified in sporadic and familial TAAD patients were more significantly more likely to include genes encoding proteins involved in contraction and adhesion of cells when compared to rare CNVs found in controls [22].

Nine genes are commonly duplicated amongst all TAAD patients harboring 16p13.1 duplications, and of these genes, *MYH11* is the most likely candidate for the predisposition to thoracic aortic disease. *MYH11* encodes the smooth muscle cell (SMC)-specific β-myosin heavy chain isoform. The monomeric unit of myosin is a multimeric complex consisting of two heavy chains associate with two pairs of light chains. These units then assemble into thick filaments that slide along adjacent α-actin-containing thin filaments to contract SMCs using the force generated by the myosin heavy chain. Prior studies on *MYH11* mutations that cause familial TAAD suggested that the mutant myosin molecules have a dominant negative effect on filament formation, supporting the hypothesis that *MYH11* mutations will disrupt SMC contractile function [18]. We demonstrated that the 16p13.1 duplications are associated with increased *MYH11* mRNA levels in aortic tissue. Data from a transgenic mouse model overexpressing an isoform of *Myh11*, SM1, similarly showed increased SM1 mRNA levels, but no increase in SM1 protein levels [29]. Studies in *C. elegans* have shown that a precise ratio of β-myosin to its cellular chaperone, UNC45, is required for proper folding of myosin and assembly into thick filaments, and an imbalance in this ratio causes the degradation of myosin heavy chain protein and dysfunction of the contractile complex [30]. Therefore, we hypothesize that overexpression of *MYH11* does not lead to increased β-myosin protein levels, possibly due to imbalance of β-myosin to its chaperone, leading to degradation of β-myosin and dysfunction of the SMC contractile unit.

One important limitation of our study is potential bias due to the differential sensitivity of SNP array platforms and quantitative PCR to detect 16p13.1 duplications, which could potentially lead to a spurious association between this CNV and TAAD. We found that the prevalence of 16p13.1 duplications was similar in discovery and replication cases, which were screened using these two different methods. In addition, the frequency of duplications in controls matched the population frequency reported in multiple previous publications. Therefore, our conclusions are unlikely to be altered significantly by the bias related to using different CNV discovery methods.

In summary, although the presence of the 16p13.1 duplication confers a risk for thoracic aortic disease, the decreased penetrance of TAAD associated with the duplication suggests that other risk factors are required for expression of the clinical phenotype [26]. The risk factors can be a genetic variant, such as another CNV, or possibly the presence of a BAV or a single gene mutation. Alternatively, other known risk factors for TAAD, such as poorly controlled hypertension, could contribute to aortic dissections in 16p13.1 duplication carriers by increasing the hemodynamic forces on the ascending aorta. With these risk factors, the presence of the 16p13.1 duplication predicts development of an acute aortic dissection at an aortic diameter less that 5.0 cm rather than a stable aneurysm when compared with thoracic aortic disease patients without the duplication. At the same time, the lack of schizophrenia and ADHD in these patients also implies that a second, and most likely different event, is required for development of neuropsychiatric disease. Further studies in larger cohorts with complete phenotypic data are needed to further define the additional genetic and environmental risk factors leading to aortic dissection or schizophrenia in patients with 16p13.1 duplications.

## Material and Methods

### Thoracic aortic aneurysm and dissection cohorts

The Institutional Review Boards at the University of Texas Health Science Center at Houston and Baylor College of Medicine approved this study. Informed consent was obtained from all study participants. A cohort of 800 patients (STAAD-1) of European descent referred for treatment of an ascending aneurysm or an ascending (Stanford type A) or descending (type B) aortic dissection was recruited (STAAD-1). The following patients were excluded from this cohort: patients less than 31 years of age; patients with aortic lesions associated with trauma, infection, aortitis, or connective tissue disorders (Marfan syndrome, Ehlers-Danlos syndrome, Loeys-Dietz syndrome); patients with a first degree relative with thoracic aortic aneurysm or dissection; patients with an isolated intramural hematoma, penetrating aortic ulcer, or pseudoaneurysm; and patients who received packed red blood cell, whole blood or platelet transfusion within 72 hours of blood collection. To select 765 ethnically matched cases, multidimensional scaling (MDS) was performed on a subset of the genome wide genotype data that were in linkage equilibrium or in low levels of linkage disequilibrium; these data came from the cases, the controls, and four HapMap populations (CEU – CEPH Utah residents with ancestry from northern and western Europe, YRI-Yoruba in Ibadan, Nigeria, JPT-Japanese in Tokyo, Japan and CBT - Han Chinese in Beijing, China) [31]. Samples that deviated by more than 4 SDs from the median of MDS components 1 and 2 were removed.

A second cohort of 242 patients meeting the same criteria was used as a replication cohort (STAAD-2). A third cohort of 130 affected probands of unrelated families with multiple members with TAAD (FTAAD cohort) who did not carry a known genetic mutation or syndrome identified as the cause of the inherited TAAD was enrolled. The probands and family members were considered affected if they had dissection of the thoracic aorta, surgical repair of an ascending aneurysm, or had dilatation of the ascending aorta greater than 4.5 cm based on echocardiographic measurements of the aortic diameter at the sinuses of Valsalva and/or the ascending aorta. Finally, a cohort of 95 patients with bicuspid aortic valve and ascending aneurysm or dissection was obtained from the GenTAC registry (BAV/TAAD cohort). No history of schizophrenia, autism, ADHD or other mental illnesses were identified in any of the above TAAD patients.

The primary controls for this study were 6809 Illumina genotypes from unaffected adults (accessions phs000092.v1.p1, phs000004.v1.p1, phs000093.v2.p2, phs000001.v2.p1 and phs000142.v1.p1), which were obtained from the Database of Genotypes and Phenotypes (dbGAP, http://www.ncbi.nlm.nih.gov/gap). As described above for the cases, MDS was used to select 5409 ethnically matched controls of European descent. The characteristics of the five control cohorts and genotypes, as well as the methods for data quality control, allele detection and genotype calling, have been described [22]. Unrelated European descent individuals from each dataset were analyzed with identical methods. Numbers of excluded genotypes were not significantly different between datasets. For the discovery cohort, 4569 controls from 4 independent datasets were analyzed. For the replication cohort, a fifth independent dataset with 840 controls was analyzed, in addition to 521 European descent healthy control DNAs from individuals who did not have cardiovascular disease. DNA was isolated from peripheral blood or buccal cells using standard methods.

### Quantitative real-time PCR for CNV assay

Quantitative PCR was performed using an ABI Prism 7900 Sequence Detection System (Applied Biosystems, Foster City, CA). Each reaction was performed in a total volume of 20 µl, containing 1× Taqman Universal PCR Master Mix, 1× RNase P Primer-Probe (VIC dye) Mix, 10 µM forward and reverse primers, 5 µM TaqMan Probe and 10 ng genomic DNA. *RNaseP* is a single copy gene present as two copies in all samples and was used as an endogenous control for normalizing the differences in input DNA. All reactions were performed in quadruplicate and repeated three times. Positive controls (samples with the 16p13.1 duplication) were randomly embedded in the TAAD samples to confirm these samples were appropriately detected as having three alleles. PCR thermocycling conditions consisted of an initial polymerase activation and DNA denaturation step at 60°C for 15 min, followed by 40 cycles of 15 s at 95°C and 1 min at 60°C. The threshold cycle (Ct) level for each tested gene was automatically determined by the Sequence Detection Software (SDS v2.1, Applied Biosystem, Foster City, CA). The copy number of the *MYH11* as well as *PDXDC1*, *C16orf45* and *ABCC1* in each tested sample was determined using Copy Caller (verson 1.0, Applied Biosystems, Foster City, CA).

### CGH using agilent oligonucleotide array

Sample DNA and reference DNA were fluorescently labeled and hybridized according to the manufacturer's protocol. Array CGH was performed with the human genome CGH microarray kit (Agilent Technologies, Santa Clara, CA). Following hybridization, slides were washed and assessed for fluorescence using an Agilent microarray scanner (Agilent Technologies, Santa Clara, CA). The scanned data were extracted using Feature Extraction 9.1.1 software (Agilent Technologies, Santa Clara, CA) and were analyzed using CGH Analytics 3.4 software (Agilent Technologies, Santa Clara, CA). Genomic copy number changes were identified with the assistance of the Aberration Detection Method 1 algorithm.

### Aortic tissue collection and analysis

Control ascending aortic tissues were obtained through the International Institute for the Advancement of Medicine (IIAM) from individuals with no known cardiovascular diseases or hypertension that died of non-vascular causes. Patients' ascending aortic tissues were obtained from STAAD patients in the operating room and transferred immediately to the laboratory for processing and freezing. Total human aortic tissue RNA was extracted with Trizol (Invitrogen, Carlsbad, CA) according to the manufacturer's protocol. Reverse transcription reactions were performed using MMLV-RT kit (Invitrogen, Carlsbad, CA) and random hexamer according to the manufacturer's protocol. For Quantitative Real-time PCR analysis of mRNA expression, TaqMan probes were purchased from Applied Biosystems and analyzed using an Applied Biosystems Prism 7900 HT Sequence Detection System (Applied Biosystems, Foster City, CA) according to the manufacturer's protocol. Experiments were performed in triplicate. Both calponin-1 (*CNN1*), which encodes another smooth muscle cell contractile protein, and *GAPDH* were used as internal controls. Formalin-fixed, paraffin-embedded aortic tissue sections from STAAD patients and controls were stained with Movat stain or immunostained with monoclonal antibody (Sigma Aldrich, St. Louis, Missouri ) for smooth muscle (SM) α-actin as described [32].

### Statistical analysis

Statistical comparisons of continuous variables between the discovery and replication cohorts were performed using the Mann-Whitney U test, while categorical variables were compared using the Chi-square test or Fisher's exact test, as appropriate. Survival curves for time to enrollment were compared using the log-rank test. Stepwise Cox proportional hazards regression was used to estimate adjusted odds ratios for predictor variables. The Breslow-Day-Tarone test was used to assess the homogeneity of the ORs between the primary and replication datasets. Statistical analysis of Q-PCR data was performed with the Mann-Whitney U test.

## Supporting Information

Figure S1 Characterization of 16p13.1 duplications in STAAD patients and comparison with other reports. (A) 16p13.1 duplications were detected in 9 out of 800 discovery cases using Illumina Human CNV370-Quad SNP arrays. The extent of 16p13.1 duplications from each of the patients is represented by a blue line with the patient sample ID on the side. The consensus region that is spanned by all CNVs is shown by a dashed red line. The common duplicated regions identified by Williams *et. al.*, Ingason *et. al.*, Ullmann *et. al.* and Hannes. *et al.* are also indicated with green lines. Below is a schematic of the 16p13.1-p12.3 region, which includes the location of genes and segmental duplications (denoted by orange, yellow and black bars). The locations of Q-PCR probes used to detect DNA duplications are indicated by colored arrows. At the bottom, the largest low copy repeats in the region (<50 kb) with high sequence homology (>98%) are shown. The arrows show directionality and the different colors denote different repeats. (B) 16p13.1 duplications identified in STAAD patients by Illumina SNP array were validated by Q-PCR assays using probes located in *PDXDC1*, *C16orf45*, *MYH11* and *ABCC1*. Patient identifiers are shown on the X-axis. The predicted copy number as detected by independent probes is shown on the Y-axis.
(TIF)

Figure S2 Illumina GenomeStudio plots of 16p13.1 duplications in STAAD patients. B allele frequencies (top) and Log R ratio values (bottom) are plotted for SNPs from each individual on chromosome 16p. The 16p13.1 duplications in patients MG7647, MG521044 and MG8233 can be identified by the deviation of heterozygous values from 0.5 to 0.67 and 0.33 in the B allele frequency plots (as indicated by the red arrows) as well as the upward shift in Log R ratios (as indicated by the blue arrows). MG5742, a STAAD patient without 16p13.1 duplication, is provided for comparison.
(TIF)

Figure S3 Agilent oligonucleotide arrays confirm 16p13.1 duplications in STAAD patients. The X-axis shows the Log2 ratios of chromosome 16p probes; the Y-axis shows the location of the probes along chromosome 16 in megabases (Mb). Regions of loss (green dots), gain (red dots) and no change (blue dots) were color-coded. The ratio of total red dots above the line to green dots below the line is greater than 1 in samples with 16p13.1 duplications (MG3392, MG5041, MG6343, MG4890 and MG4948). A negative control without 16p13.1 duplication (MG6153) is provided for comparison.
(TIF)

Figure S4 Determination of *MYH11* copy number using real-time quantitative PCR (Q-PCR). (A) Validation of *MYH11* Q-PCR assay using DNA samples from patients with sporadic thoracic aneurysms and dissections (STAAD). Predicted *MYH11* copy number values are graphed with standard errors derived from four replicate assays. Q-PCR using three different probes within the *MYH11* gene confirmed that 8 STAAD cases identified as 16p13.1 duplication carriers by microarray analysis harbor three copies of *MYH11*. Two copies of *MYH11* were confirmed in five additional STAAD cases. (B) Screening for *MYH11* duplications in healthy controls using the *MYH11* Q-PCR assay. All control samples harbored 2 copies of *MYH11* as detected by *MYH11* probe 1 and these findings were confirmed with probes 2 and 3 (data not shown). PC is a positive control with a confirmed 16p13.1 duplication (MG5041). (C) Detection of *MYH11* duplications in STAAD patients using the *MYH11* Q-PCR assay with *MYH11* probe 1. Samples S2, S4, S5, S6, S11 and S13 (original sample ID are MG4948, MG4890, MG9973, MG6983, MG9076 and MG5041) harbor 3 copies of the *MYH11* gene.
(TIF)

Table S1 Clinical characteristics of STAAD-1 and STAAD-2 cohorts.
(DOCX)

Table S2 Size of chromosome 16p13.1 duplications in 6 out of 4569 controls as detected by Illumina SNP arrays.
(DOCX)

Table S3 Large CNV second hits in individuals with 16p13.1 duplications.
(DOCX)

Table S4 Primers and probes used for determination of 16p13.1 copy number variation.
(DOCX)

Table S5 Clinical characteristics of the BAV/TAAD (GenTAC) cohort.
(DOCX)
