# Supplementary figures and images for: Recurrent Chromosome 16p13.1 Duplications Are a Risk Factor for Aortic Dissections
Source: PLoS Genet. 2011 Jun 16;7(6):e1002118. doi: 10.1371/journal.pgen.1002118 (PMC3116911; doi:10.1371/journal.pgen.1002118)

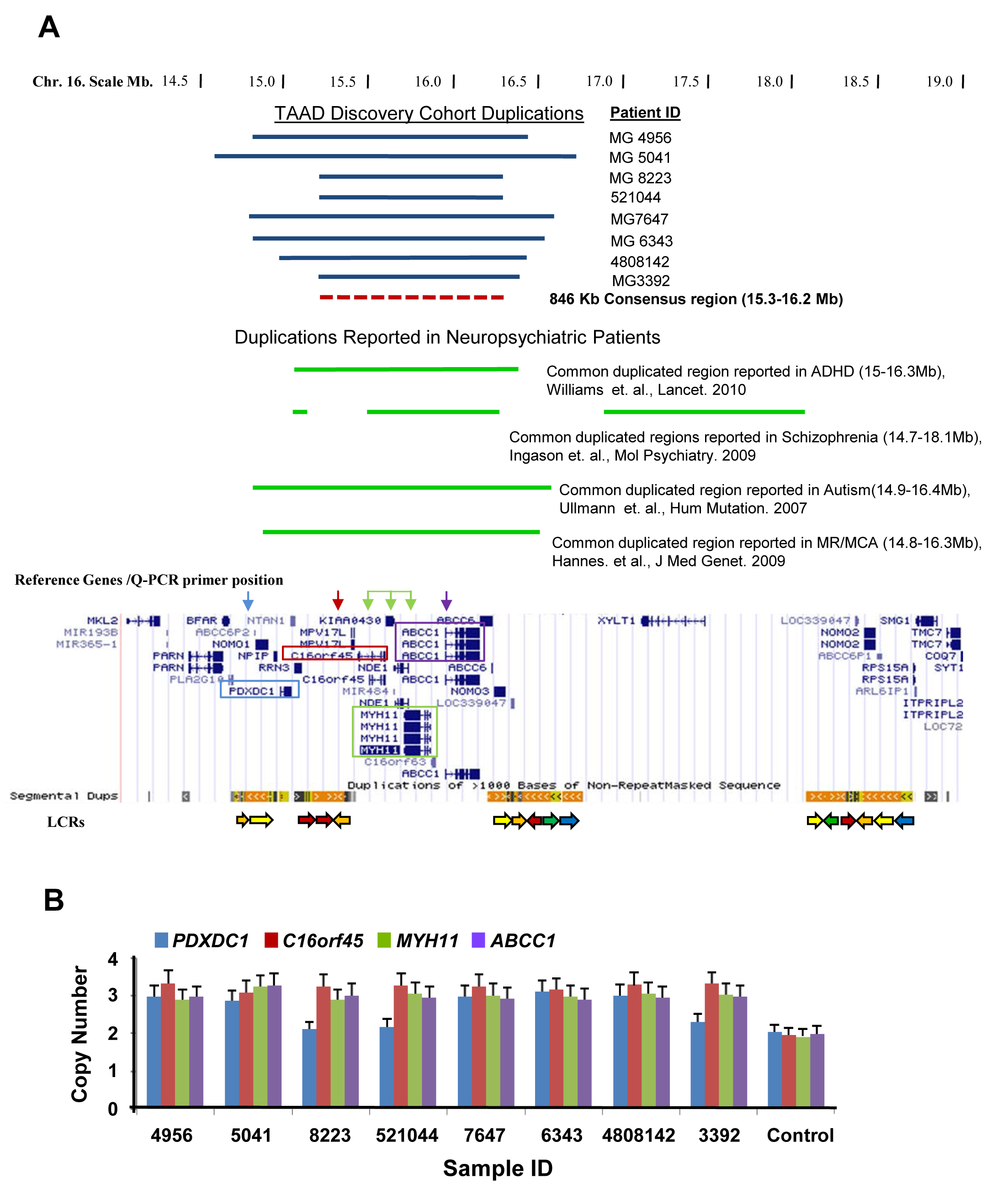

Supplement: Figure S1 — Characterization of 16p13.1 duplications in STAAD patients and comparison with other reports. (A) 16p13.1 duplications were detected in 9 out of 800 discovery cases using Illumina Human CNV370-Quad SNP arrays. The extent of 16p13.1 duplications from each of the patients is represented by a blue line with the patient sample ID on the side. The consensus region that is spanned by all CNVs is shown by a dashed red line. The common duplicated regions identified by Williams et. al., Ingason et. al., Ullmann et. al. and Hannes. et al. are also indicated with green lines. Below is a schematic of the 16p13.1-p12.3 region, which includes the location of genes and segmental duplications (denoted by orange, yellow and black bars). The locations of Q-PCR probes used to detect DNA duplications are indicated by colored arrows. At the bottom, the largest low copy repeats in the region (<50 kb) with high sequence homology (>98%) are shown. The arrows show directionality and the different colors denote different repeats. (B) 16p13.1 duplications identified in STAAD patients by Illumina SNP array were validated by Q-PCR assays using probes located in PDXDC1, C16orf45, MYH11 and ABCC1. Patient identifiers are shown on the X-axis. The predicted copy number as detected by independent probes is shown on the Y-axis. (TIF) [file pgen.1002118.s001.tif]

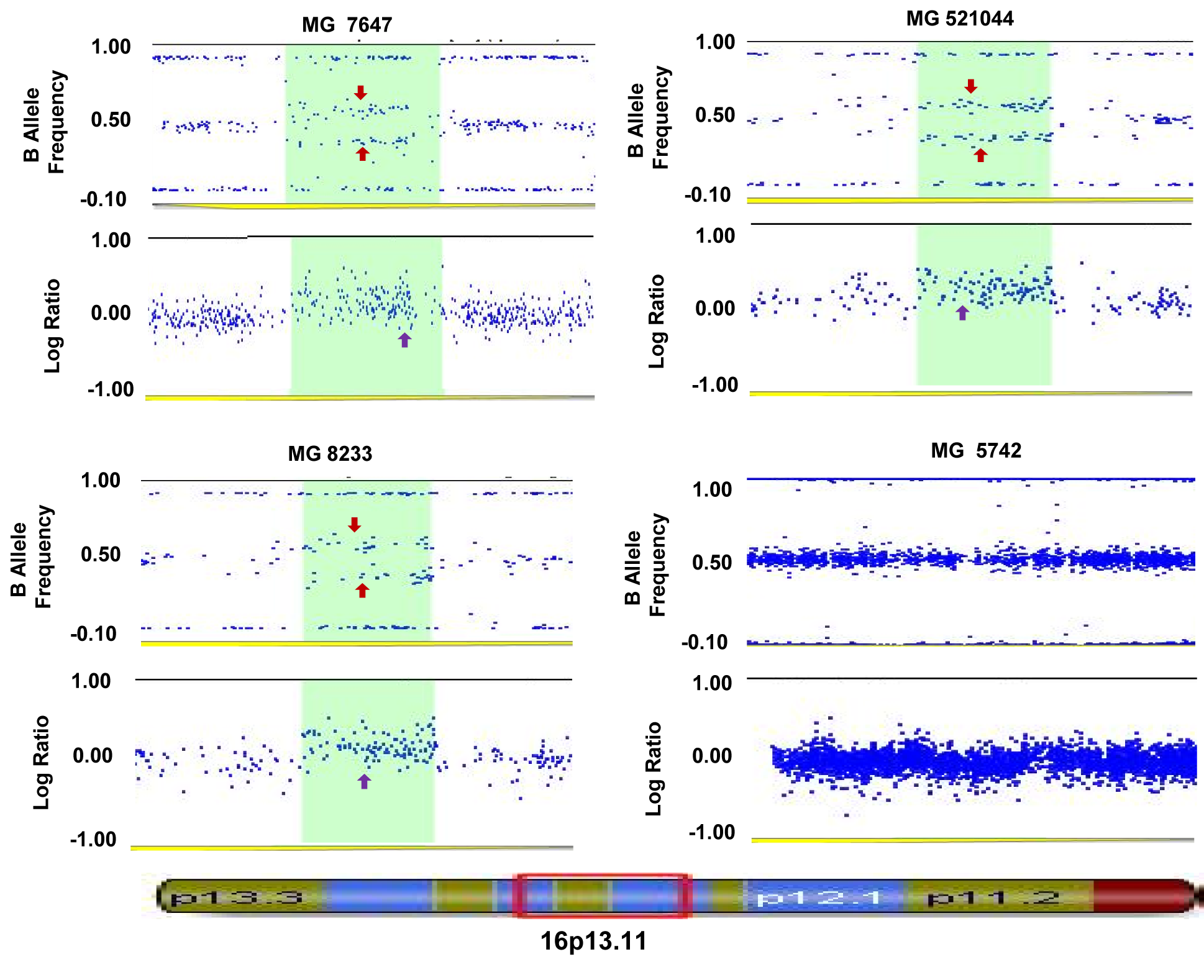

Supplement: Figure S2 — Illumina GenomeStudio plots of 16p13.1 duplications in STAAD patients. B allele frequencies (top) and Log R ratio values (bottom) are plotted for SNPs from each individual on chromosome 16p. The 16p13.1 duplications in patients MG7647, MG521044 and MG8233 can be identified by the deviation of heterozygous values from 0.5 to 0.67 and 0.33 in the B allele frequency plots (as indicated by the red arrows) as well as the upward shift in Log R ratios (as indicated by the blue arrows). MG5742, a STAAD patient without 16p13.1 duplication, is provided for comparison. (TIF) [file pgen.1002118.s002.tif]

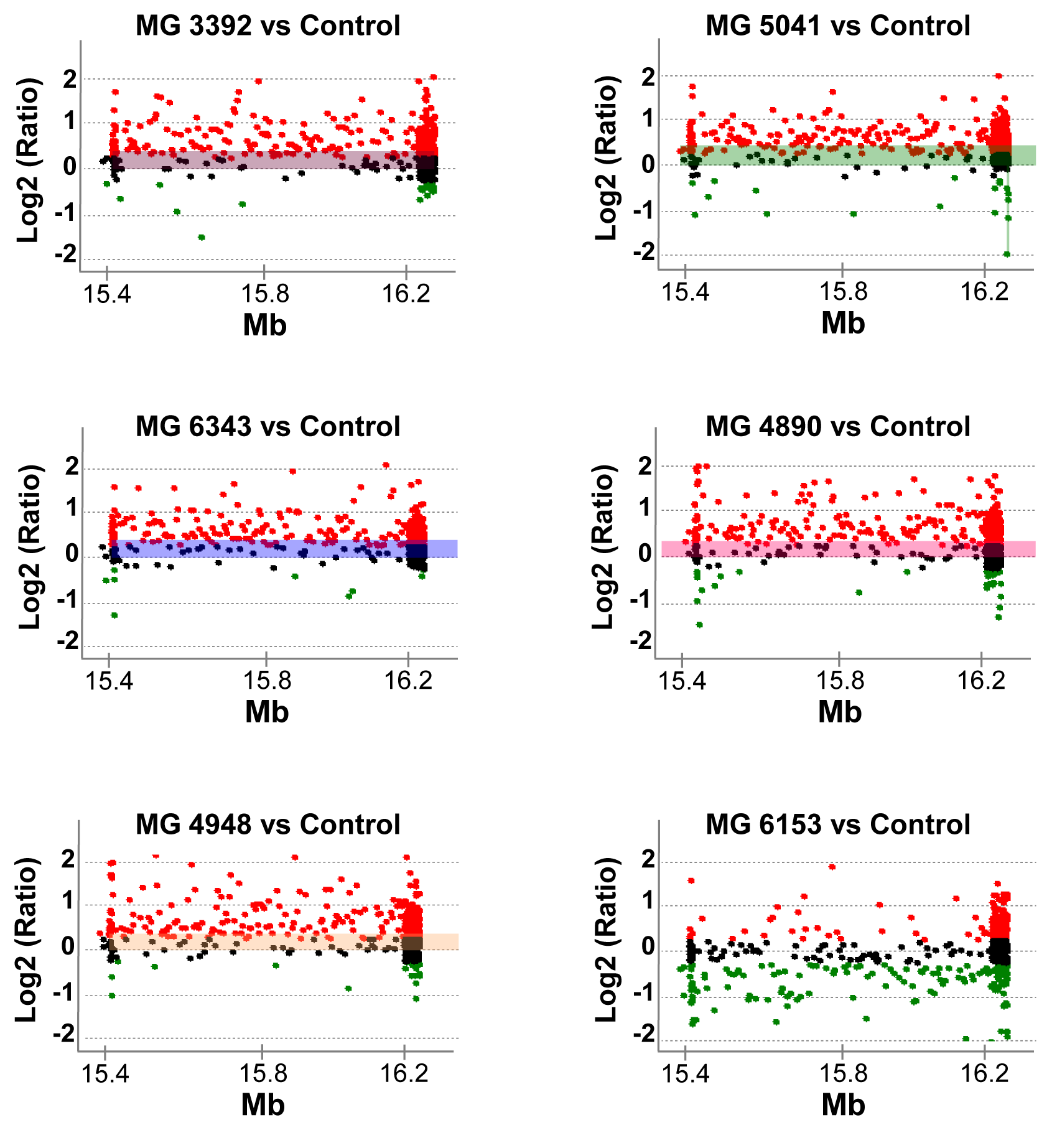

Supplement: Figure S3 — Agilent oligonucleotide arrays confirm 16p13.1 duplications in STAAD patients. The X-axis shows the Log2 ratios of chromosome 16p probes; the Y-axis shows the location of the probes along chromosome 16 in megabases (Mb). Regions of loss (green dots), gain (red dots) and no change (blue dots) were color-coded. The ratio of total red dots above the line to green dots below the line is greater than 1 in samples with 16p13.1 duplications (MG3392, MG5041, MG6343, MG4890 and MG4948). A negative control without 16p13.1 duplication (MG6153) is provided for comparison. (TIF) [file pgen.1002118.s003.tif]

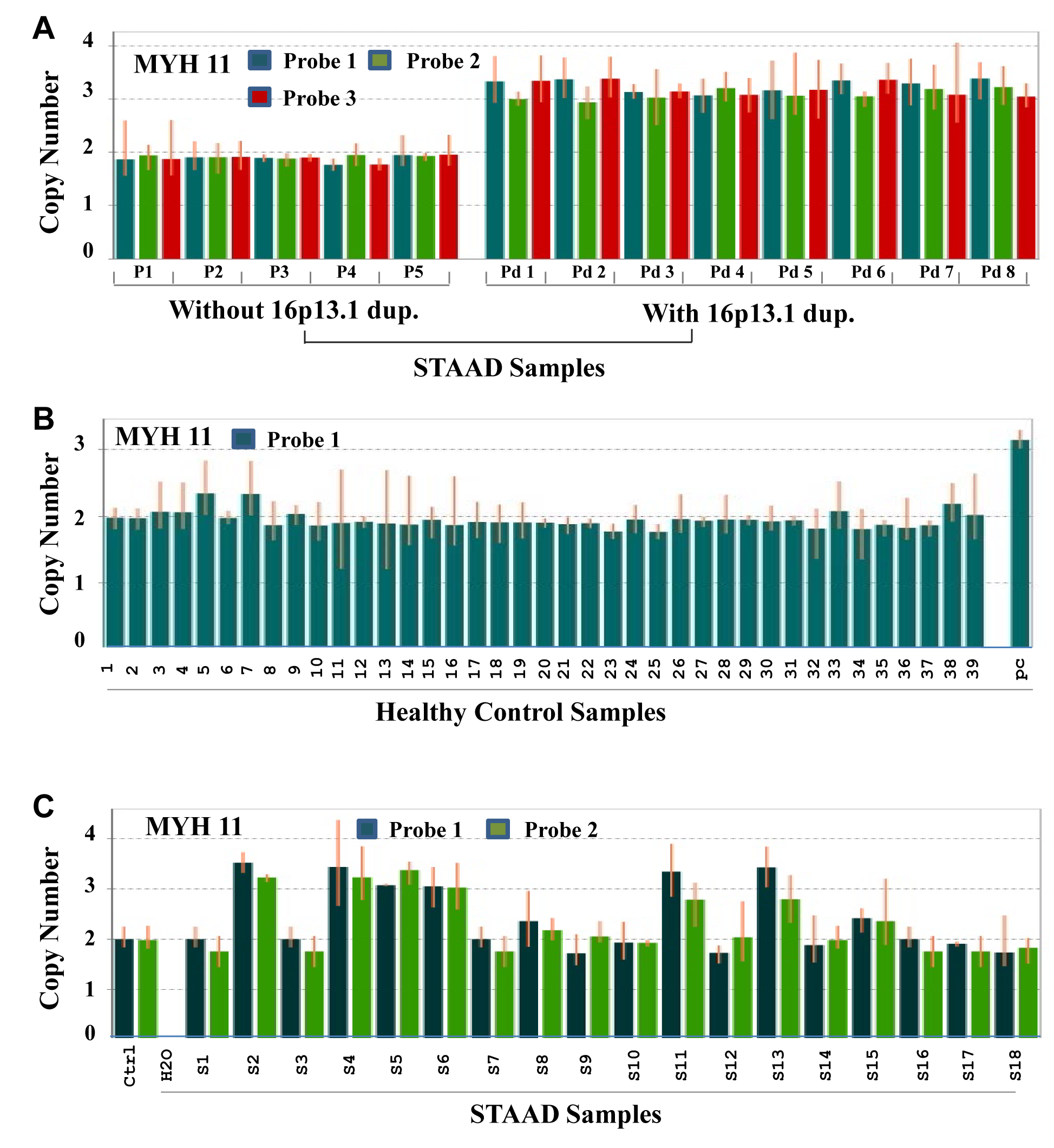

Supplement: Figure S4 — Determination of MYH11 copy number using real-time quantitative PCR (Q-PCR). (A) Validation of MYH11 Q-PCR assay using DNA samples from patients with sporadic thoracic aneurysms and dissections (STAAD). Predicted MYH11 copy number values are graphed with standard errors derived from four replicate assays. Q-PCR using three different probes within the MYH11 gene confirmed that 8 STAAD cases identified as 16p13.1 duplication carriers by microarray analysis harbor three copies of MYH11. Two copies of MYH11 were confirmed in five additional STAAD cases. (B) Screening for MYH11 duplications in healthy controls using the MYH11 Q-PCR assay. All control samples harbored 2 copies of MYH11 as detected by MYH11 probe 1 and these findings were confirmed with probes 2 and 3 (data not shown). PC is a positive control with a confirmed 16p13.1 duplication (MG5041). (C) Detection of MYH11 duplications in STAAD patients using the MYH11 Q-PCR assay with MYH11 probe 1. Samples S2, S4, S5, S6, S11 and S13 (original sample ID are MG4948, MG4890, MG9973, MG6983, MG9076 and MG5041) harbor 3 copies of the MYH11 gene. (TIF) [file pgen.1002118.s004.tif]
