# Supplementary material for: Recurrent Chromosome 16p13.1 Duplications Are a Risk Factor for Aortic Dissections
Source: PLoS Genet. 2011 Jun 16;7(6):e1002118. doi: 10.1371/journal.pgen.1002118 (PMC3116911; doi:10.1371/journal.pgen.1002118)
Supplement: Table S1 — Clinical characteristics of STAAD-1 and STAAD-2 cohorts. (DOCX) [file pgen.1002118.s005.docx]

**Table S1.** Clinical characteristics of STAAD-1 and STAAD-2 cohorts.

| **Variable** | **STAAD-1 Cohort**  **(n =765 )** | **STAAD-2 Cohort**  **(n = 242)** | ***P*-value** |
| --- | --- | --- | --- |
| Age (years) | 65 (55-73, min=31, max=94) | 65 (55-73, min=31, max=89) | 0.8 |
| Male gender | 500 (65.4%) | 161 (66.8%) | 0.6 |
| Height (cm) | 175 (168-183, min=140, max=201) | 175 (166.5-182, min=145, max=201) | 0.5 |
| Weight (kg) | 82 (70-97, min=38, max=164) | 84 (73-100, min=39, max=195) | 0.3 |
| Body surface area (m^2^) | 2.02 (1.83-2.23, min=1.31, max=3.00) | 2.05 (1.86-2.24, min=1.28, max=3.31) | 0.4 |
| Hypertension | 589 (77.0%) | 206 (85.5%) | 0.004 |
| Smoking (past or present) | 470 (61.4%) | 133 (55.2%) | 0.1 |
| Dissection | 364 (47.6%) | 115 (47.7%) | 1.0 |
| Type A dissection | 224^*^ (29.3%) | 68^†^ (28.2%) | 0.8 |
| Type B dissection | 144^*^ (18.8%) | 52^†^ (21.6%) | 0.4 |
| Ascending aortic aneurysm  (no aortic dissection) | 401 (52.4%) | 126 (52.3%) | 1.0 |
| Bicuspid aortic valve | 147 (19.2%) | 46 (19.1%) | 1.0 |

^*^4 patients with both type A and B dissections

^†^5 patients with both type A and B dissections
