# Supplementary material for: Recurrent Chromosome 16p13.1 Duplications Are a Risk Factor for Aortic Dissections
Source: PLoS Genet. 2011 Jun 16;7(6):e1002118. doi: 10.1371/journal.pgen.1002118 (PMC3116911; doi:10.1371/journal.pgen.1002118)
Supplement: Table S2 — Size of chromosome 16p13.1 duplications in 6 out of 4569 controls as detected by Illumina SNP arrays. (DOCX) [file pgen.1002118.s006.docx]

**Table S2.** Size of chromosome 16p13.1 duplications in 6 out of 4569 controls as detected by Illumina SNP arrays.

*dup: duplication.
