# Supplementary material for: Recurrent Chromosome 16p13.1 Duplications Are a Risk Factor for Aortic Dissections
Source: PLoS Genet. 2011 Jun 16;7(6):e1002118. doi: 10.1371/journal.pgen.1002118 (PMC3116911; doi:10.1371/journal.pgen.1002118)
Supplement: Table S5 — Clinical characteristics of the BAV/TAAD (GenTAC) cohort. (DOCX) [file pgen.1002118.s009.docx]

**Table S5.** Clinical characteristics of the BAV/TAAD (GenTAC) cohort.

| Variable |  |
| --- | --- |
| Age (years) (n=95) | 53.0 (36.4-57.8, min=8, max=74) |
| Male gender | 75 (78.9%) |
| Height (cm) (n=84) | 178 (165-182, min=131, max=200) |
| Weight (kg) (n=86) | 86.9 (72.1-99.0, min=29, max=145) |
| Body surface area (m^2^) (n=84) | 2.09 (1.86-2.20, min=1.07, max=2.69) |
| Dissection | 8 (8.4%) |
| Type A dissection | 6 (6.3%) |
| Ascending aortic aneurysm (no dissection) (n=80) | 74 (77.9%) |
| TAAD surgery | 59 (62.1%) |
| Surgery age (n=39) | 50.1 (28.4-57.5, min=5, max=69) |
